# Heterogeneity of HLA-G Expression in Cancers: Facing the Challenges

**DOI:** 10.3389/fimmu.2018.02164

**Published:** 2018-09-27

**Authors:** Aifen Lin, Wei-Hua Yan

**Affiliations:** ^1^Biological Resource Center, Taizhou Hospital of Zhejiang Province, Wenzhou Medical University, Linhai, China; ^2^Medical Research Center, Taizhou Hospital of Zhejiang Province, Wenzhou Medical University, Linhai, China

**Keywords:** HLA-G, heterogeneity, cancer, immune evasion, therapy target

## Abstract

Phenotypic heterogeneity has been observed in most malignancies, which represents a considerable challenge for tumor therapy. In recent decades, the biological function and clinical significance of the human leukocyte antigen (HLA)-G have been intensively explored. It is now widely accepted that HLA-G is a critical marker of immunotolerance in cancer cell immune evasion and is strongly associated with disease progress and prognosis for cancer patients. Moreover, it has recently been emphasized that the signaling pathway linking HLA-G and immunoglobulin-like transcripts (ILTs) is considered an immune checkpoint. In addition, HLA-G itself can generate at least seven distinct isoforms, and intertumor and intratumor heterogeneity of HLA-G expression is common across different tumor types. Furthermore, HLA-G heterogeneity in cancers has been related to disease stage and outcomes, metastatic status and response to different therapies. This review focuses on the heterogeneity of HLA-G expression in malignant lesions, and clinical implications of this heterogeneity that might be relevant to personalized treatments are also discussed.

## Introduction

Cancer is a very complex and heterogeneous disease that involves a broad range of mixed cells with distinct features. Tumor cells not only vary in morphology and phenotype but also, in genomes, transcriptomes, epigenomes and proteomes (1). Diversity among tumor cells termed heterogeneity can be observed between tumor cells within a tumor (intratumor heterogeneity). However, tumor heterogeneity is also common between the primary tumor and metastatic tumors, between metastases from the same patient, and between tumors of the same histotype from different patients (intertumor heterogeneity) (2). Tumor heterogeneity is now widely acknowledged to influence tumor cell characteristics such as growth, survival, metastasis and response to various therapies and immune evasion. Consequently, tumor heterogeneity presents a formidable obstacle in cancer treatment (3).

Human leukocyte antigen (HLA)-G, a non-classical HLA class I molecule, encompasses at least four membrane-bound (mHLA-G, HLA-G1~HLA-G4) and three soluble (sHLA-G, HLA-G5~HLA-G7) isoforms resulting from alternative splicing of in its primary mRNA. HLA-G expression was initially observed in extravillous cytotrophoblasts and is considered to play important roles in maintenance of fetal-maternal immune tolerance (4, 5). In addition to extravillous cytotrophoblasts, HLA-G expression is restricted to a few healthy adult tissues, including the cornea, thymic medulla and pancreatic islets (6–8). However, HLA-G expression can be switched on in various pathological conditions such as cancers, viral infection, organ transplantation, and autoimmune and inflammatory diseases (9).

Though the functions of HLA-G were first explored in reproductive immune regulation, its pre-clinical significance in tumor biology has been intensively investigated (10). In the context of tumor biology, Paul et al. (11) first reported that HLA-G expression was specifically observed in melanoma lesions but, absent in the adjacent non-tumorous tissues. This finding has been solidified by numerous subsequent studies with thousands of samples from more than thirty different types of tumors. It is now well established that HLA-G expression in cancers is highly related to immune suppressive microenvironments, advanced tumor stage, and poor therapeutic responses and prognosis (12, 13).

In the context of heterogeneity of HLA-G expression in cancers, the degree of HLA-G expression and the isoform profiles vary dramatically among tumor types and patients, within tumors of the same type, and between the primary tumor and metastases (10). In clinical settings, the relevance of HLA-G heterogeneity in therapeutic and immune responses, tumor progression and prognosis has been documented in a body of previous studies (14, 15). Herein, we reviewed the literature on HLA-G heterogeneity in cancers, and the clinical implications of this heterogeneity that might be relevant to personalized treatments were also discussed.

## Diversity of the HLA-G isoforms

Unlike the case for classical HLA class I counterparts, at least seven HLA-G isoforms, including four membrane-bound (HLA-G1, -G2, -G3, and -G4) and three soluble (HLA-G5, -G6, and -G7) isoforms, can be generated by alternative splicing of HLA-G primary transcripts (16). Moreover, proteolytic cleavage of cell surface HLA-G1 by metalloproteinases (MMPs) such as MMP-2 results in another soluble isoform called shedding HLA-G1 (17). Different HLA-G isoforms are distinguished by the number of extracellular immunoglobulin-like domains and whether intronic sequence encoded residues are included or not; however, all seven reported HLA-G isoforms have the extracellular α1 domain (18). The conformation of HLA-G1 and -G5 is similar to that of the classical HLA class I antigens, which contain three extracellular domains including α1, α2, and α3 non-covalently bound to β_2_-macroglobulin (β_2_m). The antigen presenting peptide-binding cleft is formed by the α1 and α2 domains in HLA-G1 and HLA-G5 molecules (19).

**Figure 1:**
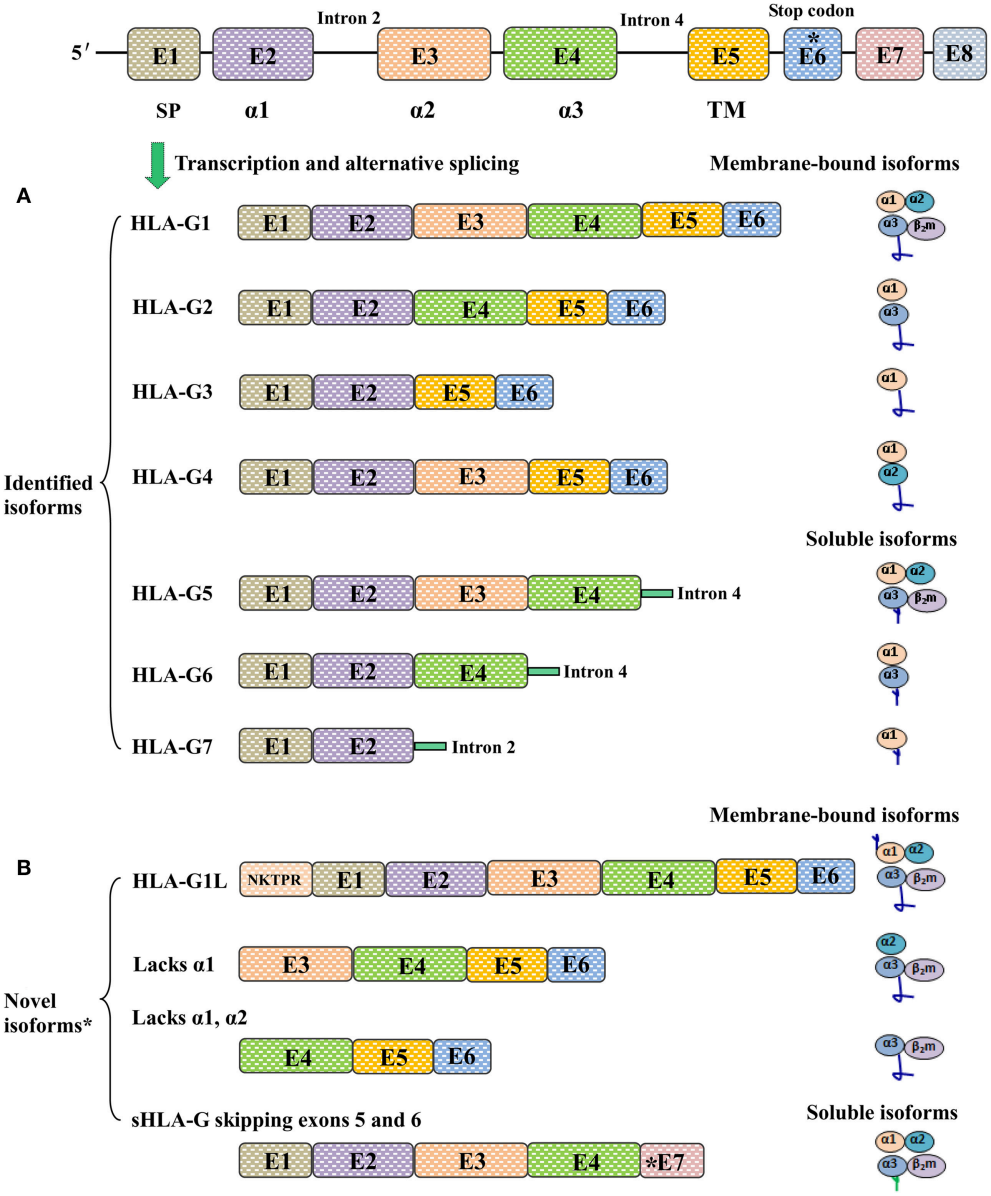
Different HLA-G isoforms generated by alternative splicing of HLA-G mRNA. **(A)** Seven identified HLA-G isoforms including four membrane-bound (HLA-G1, -G2, -G3, -G4) and three soluble (HLA-G5, -G6, -G7) molecules. The extracellular structures of HLA-G1 and HLA-G5 contain α1, α2, and α3 domains; HLA-G2 and HLA-G6 contain α1 and α3 domains; HLA-G3 contains α1 domains; HLA-G4 contains α1 and α2 domains; HLA-G7 contains an α1 domain linked to two amino acids encoded by intron 2. **(B)** Novel HLA-G isoforms predicted by Tronik-Le Roux et al. (20)*. N-terminal ends including the additional five amino acids (NKTPR) in HLA-G1L, and potential isoforms contain α2 and α3 domains or only the α3 domain. Novel soluble HLA-G isoforms generated by skipping exons 5 and 6, and with distinct C-terminal ends.

Other HLA-G isoforms lack one or two extracellular domains (α2, or α3, or both) and are smaller than HLA-G1. The extracellular structure of HLA-G2 contains α1 and α3 domains but lacks the α 2 domain; HLA-G3 contains only an α1 domain, and both α 2 and α 3 are deleted; HLA-G4 contains α1 and α2 domains, while the α 3 domain is deleted. The C terminals of soluble HLA-G isoforms including HLA-G5 (counterpart of HLA-G1) and HLA-G6 (counterpart of HLA-G2) are encoded by intron 4. HLA-G7 consists of only an α1 domain linked to two amino acid residues encoded by intron 2 (10). Notably, a study by Tronik-Le Roux et al. (20) recently reported that previously undescribed novel HLA-G isoforms were predicted by transcriptome analysis in renal cancer lesions. However, more efforts should be carried out in the development of new antibodies to identify these new isoforms and in evaluating their biological functions and clinical relevance (Figure 1).

Among the HLA-G isoforms reported to date, HLA-G1 and HLA-G5 have been studied more extensively than the others due to the available antibodies (12). However, available antibodies for HLA-G detection have a limited ability to discriminate different isoforms, which has made it difficult to test the functional importance of a specific isoform. Currently available antibodies for HLA-G detection and their specificities are detailed in Supplementary Table 1.

In addition to the different isoforms, the structure of HLA-G is even more complex, since these isoforms can be presented as homo- and hetero-multimers resulting from intermolecular disulphide bonds by Cys^42^ or Cys^147^ in the α1 or α2 domain, respectively. Furthermore, HLA-G molecules can be ubiquitinated, glycosylated and nitrated by post-translational modifications (21–23). Finally, HLA-G has been reported as a part of exosomes (24).

## HLA-G-mediated immune suppression

HLA-G has comprehensive suppressive functions exerted in multiple steps to impair anti-tumor immune responses by interacting with receptors expressed on immune cells. To date, several receptors for HLA-G have been identified, such as CD85j/immunoglobulin-like transcript 2 (ILT2), CD85d/ILT4, and CD158d/killer cell immunoglobulin-like receptor 2DL4 (KIR2DL4). Moreover, CD8 and CD160 have also been reported to bind HLA-G (25). Among these receptors, ILT2 is present on all monocytes and B lymphocytes, and on subsets of dendritic cells (DCs), myeloid derived suppressive cells (MDSCs), natural-killer (NK) cells and T cells (26). ILT4 is mainly expressed on DCs and monocytes, neutrophils and MDSCs (27–29); KIR2DL4 has been found predominately in decidual NK cells (30). Other receptors such as CD160 are expressed by subsets of CD8^+^, CD4^+^, Tγ/δ and CD56^dim^ NK cells, and by activated endothelial cells and intestinal intraepithelial cells. CD8 is a hallmark of cytotoxic T cells and is also expressed by some NK cells (26).

**Figure 2:**
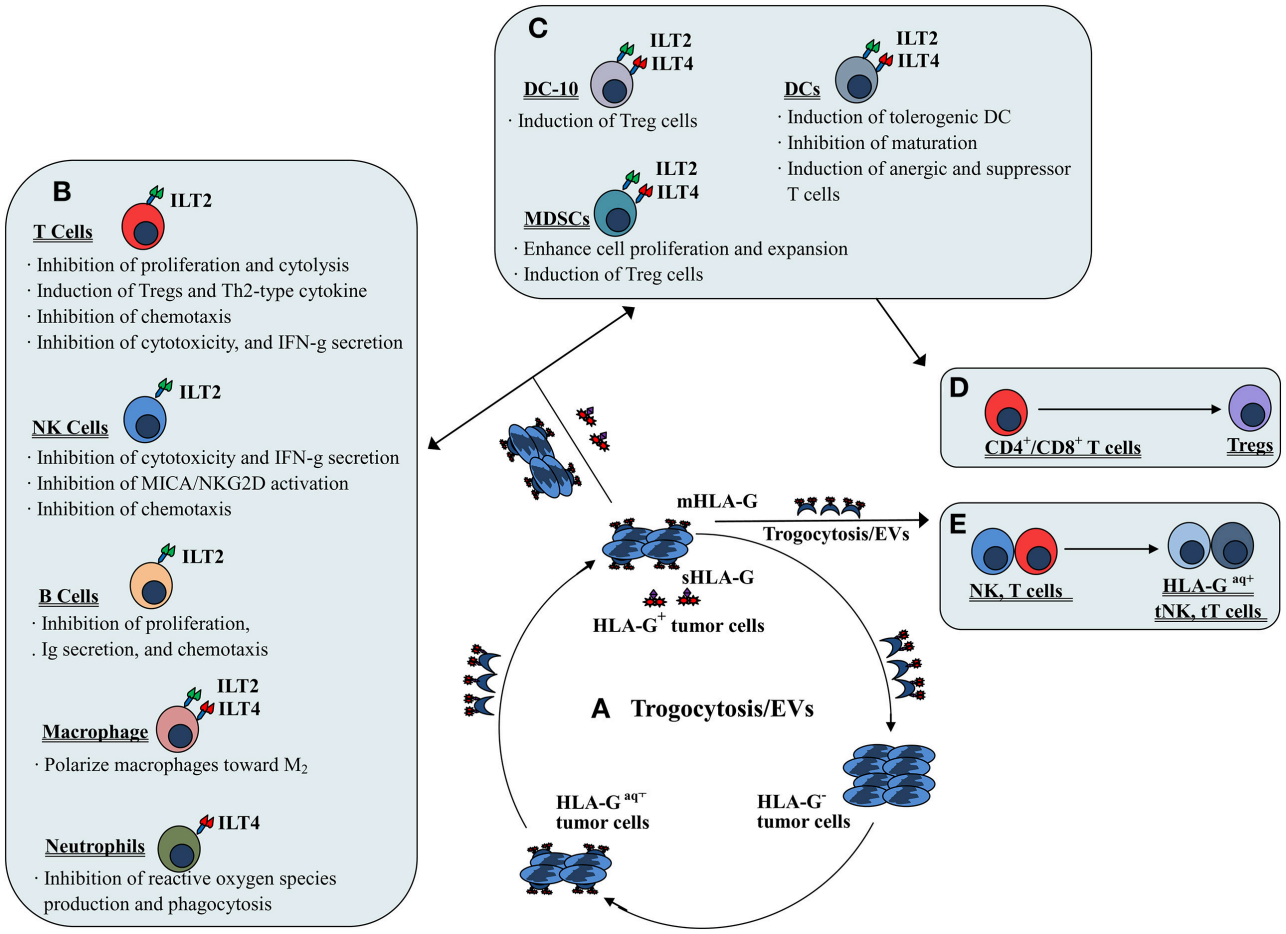
Mechanisms of both membrane-bound and soluble HLA-G-mediated immune suppression in tumor immune evasion. **(A)** Dynamic transferring of HLA-G by trogocytosis (membrane-bound HLA-G) and/or extracellular vesicles (EVs, both membrane-bound and soluble HLA-G) between HLA-G^+^ and HLA-G^−^ tumor cells. **(B)** Direct HLA-G-mediated immunosuppressive effects through engagement of inhibitory receptors (ILT2 and/or ILT4) expressed by immune cells such as T cells, NK cells, B cells, macrophages and neutrophils. **(C)** Indirect HLA-G-mediated immunosuppressive effects by induction of immune suppressive or regulatory cells such as tolerogenic DCs and MDSCs, which induce **(D)** CD4^+^/CD8^+^ T cells to become regulatory T cells (Tregs). **(E)** Immune effectors such as NK cells and T cells efficiently act as suppressor cells when they acquire HLA-G from HLA-G^+^ tumor cells or HLA-G^+^ immune cells via the process of trogocytosis and/or EVs.

The mechanisms involved in HLA-G/receptor (particularly ILT2 and ILT4) mediated immune suppression have been documented in previous studies and include impairment of immune cell proliferation, differentiation, cytotoxicity, cytokine secretion and chemotaxis; and induction of regulatory cells and MDSCs or M2 type macrophages (31–33) (Figure 2). ILT2 and ILT4 have four tandem immunoglobulin-like extracellular domains (D1~D4), a transmembrane region of 23 amino acids and three immunoreceptor tyrosine-based inhibitory motifs (ITIMs) in their cytoplasmic tails (34). The extracellular domains (D1~D2) of ILTs bind to the α3 domain in the HLA I molecule. Among HLA I family members, HLA-G binds ILTs with the highest affinity. Structural analysis showed that ILT4 could recognize both HLA-G associated with β_2_m and free HLA-G heavy chains, whereas ILT2 only recognized HLA-G associated with β_2_m. The HLA-G binding affinities of the ILT2 and ILT4 are also different; ILT4 binds HLA-G with higher affinity than ILT2 due to the different recognition specificities. Previous studies revealed that residues Tyr^36^ and Arg^38^ in ILT4 recognize the 195–197 loop, whereas the Tyr^38^ and Tyr^76^ locations of ILT2 bind Phe^195^ in the α3 domain of the HLA-G molecule (35). Interaction of HLA-G with ILTs is a critical step for HLA-G-mediated immune regulation. This HLA-G/ILTs interaction causes phosphorylation of ITIMs and recruits protein tyrosine phosphatase Src homology 2 (SH2) domain-containing proteins such as SHP-1 and SHP-2, which initiates the inhibitory signaling cascade (36, 37).

Moreover, it has also been reported that HLA-G can up-regulate tumor-promoting agents such as MMPs (38). Because of these functions, neoexpression of the HLA-G molecule on tumors favors carcinogenesis and tumor progression. Fortunately, HLA-G down-regulation with RNA interference or antibody blockade can recover the functions of immune effectors and prevent tumor reoccurrence, raising the possibility that HLA-G/ILTs interaction blockade could be a potential immuno-therapeutic strategy for cancer treatment (32, 39, 40).

### Direct HLA-G-mediated immune suppression through inhibitory receptor engagement

Direct immunosuppressive effects induced by HLA-G could occur through the engagement of inhibitory receptors expressed by various immune cells such as NK cells, T cells, B cells, neutrophils, macrophages, and DCs.

By interaction with the receptor ILT2, HLA-G could directly inhibit the immune function of NK cells, T cells and B cells. Inhibition of NK cell lysis against targets by HLA-G expression has been observed for isolated decidual NK cells, peripheral blood and NK cell lines (41, 42). HLA-G expression was also found to inhibit xenogeneic cytotoxicity and rolling adhesion of activated human NK cells to porcine endothelial cells (43, 44). Furthermore, HLA-G/ILT2 mediated inhibitory signals have been shown to mitigate major histocompatibility complex class I-related chain A (MICA)/ natural-killer group 2 member D (NKG2D)-induced activation (45). Our previous studies revealed that both the HLA-G1 and HLA-G5 isoforms could suppress NK cell cytolysis in a manner dependent on the HLA-G expression, and HLA-G1 and HLA-G5 isoforms had an additive effect on NK cytolysis suppression (46, 47). Other studies also reported that HLA-G expression in tumor cell lines such as ovarian carcinoma, hepatocellular carcinoma (HCC), glioma, and renal cell carcinoma could be protective against NK cytolysis. These studies also revealed that the inhibition could be reversed by blocking HLA-G antigens or its receptor with respective specific antibodies (48–51). For T cells, HLA-G could inhibit the CD4^+^ T cell alloproliferative response and cytolytic functions of CD8^+^ and Vγ9Vδ2 T cells through ILT2 (52–54). Regarding B cells, Naji et al. (55) demonstrated that the sHLA-G/ILT2 pathway could induce B cell G0/G1 cycle arrest. Consequently, the proliferation, differentiation, and immunoglobulin (Ig) secretion of B cells was suppressed.

Moreover, sHLA-G/ILT2 interaction impairs expression and functions of chemokine receptors in T cells, NK cells and B cells (31). sHLA-G could down-regulate the expression of CCR2, CXCR3, and CXCR5 on CD4^+^ T cells, and CXCR3 on CD8^+^ T cells and Vγ9Vδ2 T cells. Additionally, sHLA-G could impair the CD4^+^ T cell chemotaxis response to CCL2, CCL8, CXCL10, the CXCL11, CD8^+^ T cells response to CXCL10 and CXCL11, and the Vγ9Vδ2 T cells response to CXCL10 and CXCL11 (56). For NK cells, sHLA-G/ILT2 binding could down-modulate CXCR3, CX3CR1, and CCR2 expression and binding to their specific ligands such as CCL2 and CXCL10 (26). Moreover, HLA-G could decrease germinal center B cell CXCR5 expression and dampen the chemotaxis of those cells to their chemokines (57).

The immune suppressive functions of HLA-G on neutrophils have been demonstrated in a study by Baudhuin et al. (27) who found that HLA-G5/ILT4 engagement impairs neutrophil phagocytosis and reactive oxygen species production. For DCs, the expression of HLA-G has been observed to inhibit DC maturation and differentiation, and to disturb their cross-talk with NK cells. A study by Liang et al. (58) revealed that HLA-G/ILT4 interaction plays a critical role in DC differentiation modulation through the IL-6-STAT3 signaling pathway.

When HLA-G binds ILT2 and ILT4 expressed on macrophages, it not only induces macrophage differentiation toward an M2 phenotype but also enhances macrophage IL-6 and CXCL1 secretion, which could inhibit the production of interferon-γ (IFN-γ) by T cells (33). Notably, Barkal et al. (59) recently released important findings that the MHC class I component β_2_m expressed by cancer cells could directly prevent malignant cells from performing phagocytosis through ILT2. However, knowledge of the biological relevance of HLA-G expression for the regulation of macrophage functions is very limited. Therefore, more studies to explore the significance of interactions between HLA-G and macrophage receptors such as ILTs are needed.

### Indirect HLA-G-mediated immune suppression through suppressor/regulatory cells

Indirect immunosuppressive effects of HLA-G could be mediated by the induction of immune suppressive or regulatory cells such as regulatory T cells (Tregs), tolerogenic DCs and MDSCs.

HLA-G1-expressing antigen presenting cells (APCs) have been found to induce CD4^+^ T cell anergy and differentiation into suppressive cells. Furthermore, HLA-G-induced tolerogenic DCs can induce the generation of CD4^+^CD25^+^CTLA-4^+^ and CD8^+^CD28^+^ regulatory T cells (25, 60). The tolerogenic DC sub-population DC-10s, expresses high levels of HLA-G and can potently induce adaptive type 1 regulatory T cells (Tr1) through the HLA-G/ILT4 signaling pathway (28).

The evidence for a role of HLA-G in MDSC proliferation and function modulation has been obtained mainly from experiments with murine models. The HLA-G/ILT2 signaling pathway is considered to play an important role in the expansion of CD11b^+^Gr1^+^ MDSCs in ILT2 transgenic C57BL/6 mice and is directly related to the long-term survival of skin allografts (61). HLA-G5 expression has been observed to favor the expansion of CD11b^+^Ly6G^+^ MDSCs in a tumor-bearing Balb/c murine model with the murine mammary carcinoma cell line 4T1 (62). Similar findings, such as the fact that HLA-G and paired immunoglobulin-like receptor B (PIR-B, murine homolog of human ILT receptors) engagement expanded the population of CD11b^+^Gr1^+^PIR-B^+^ MDSCs in an M8-HLA-G1 (human melanoma cell line) tumor-bearing mouse model, have also been reported (32). In addition to the murine models, a recent study by Köstlin et al. (29) revealed that HLA-G/ILT4 interaction could promote MDSC accumulation and suppressive activity during human pregnancy.

### HLA-G-mediated immune suppression through trogocytosis/extracellular vesicles

In addition to HLA-G expression and interactions with receptor, HLA-G mediated immune suppression by intercellular transfer mechanisms such as trogocytosis or extracellular vesicles has gained considerable attention in recent years.

Trogocytosis is a rapid process of transferring cell membrane fragments containing molecules from one cell to another during cell-to-cell contact (63). Immune cells such as activated NK cells, T cells and monocytes can rapidly acquire membrane fragments containing functional HLA-G from other cells (i.e., HLA-G+ immune or tumor cells) in their vicinity by the process of trogocytosis. The acquired HLA-G molecule can then immediately reverse the functional phenotype of the recipient from effector to regulatory cells (64). In this scenario, when activated NK cells acquired HLA-G1 from M8-HLA-G1 tumor cells, NK-HLA-G1acq+ cells lost their cytolytic functions and ceased cell proliferation. Moreover, NK-HLA-G1acq+ cells behaved as suppressor cells capable of suppressing the cytolytic functions other NK cells (65). In other immune cells such as T cells and monocytes, acquisition of HLA-G from tumor cells or APCs can also rapidly reverse the phenotype from effector to regulatory (66). In clinical settings, CD25–FoxP3– T cells can acquire HLA-G from HLA-G+ malignant plasma cells in multiple myeloma patients. Functionally, these HLA-G+ T cells act as Tregs with inhibitory functions similar to those of natural Tregs (67). Furthermore, intercellular transfer of HLA-G from allogeneic as well as from autologous HLA-G positive tumor cells to HLA-G negative tumor cells via trogocytosis has been demonstrated (68).

Extracellular vesicles (EVs) are highly heterogeneous small phospholipid bilayer vesicles that are released by most normal and malignant cells (69). EVs harbor different types of genetic materials and proteins. HLA-G-bearing EVs were first observed in culture supernatants of an HLA-G+ melanoma cell line (Fon), and later in ascites and pleural exudates from cancer patients (21, 70). Moreover, high levels of HLA-G-bearing EVs were observed to be positively associated with disease progression in advanced breast cancer patients undergoing neoadjuvant chemotherapy (71). A recent study by Grange et al. (72) reported that HLA-G-bearing EVs derived from renal cancer cells could impair monocyte-derived DC differentiation and T cell immune responses, indicating the potential of HLA-G-bearing EVs to modulate functions of both the innate and adaptive immune systems. These findings highlighted that the effects of HLA-G-mediated tumor immune evasion can be extended to HLA-G-negative tumor cells by the pathways of trogocytosis and/or extracellular vesicles.

Given the abovementioned immune suppressive functions of HLA-G in cancer immunology, the HLA-G/ILTs signaling pathway has been recently recognized as a new immune checkpoint in addition to other immune checkpoints such as cytotoxic T lymphocyte-associated protein 4 (CTLA-4)/B7 and programmed cell death protein-1 (PD-1)/PD-L1 (40). Functionally, CTLA4/B7 interactions specifically inhibit T cell responses during T cell priming by competing with CD28 for the B7 receptor and thereby preventing CD4^+^ T cell activation. PD1/PDL1 interactions have prominent roles in interfering with T cell receptor (TCR) signaling and result in dysfunction and exhaustion of activated T cells (73). Thus, blocking either CTLA-4 or PD-1 has emerged as a promising anti-cancer strategy. However, it is evident from large clinical trials that only a fraction of patients respond with durable remission or are cured, while many patients will relapse (74). One can hypothesize that different responses to checkpoint inhibitor therapy could be a consequence of heterogeneous intra- and inter-tumor expression of different kinds of checkpoints, though data on the expression status of ILTs and CTLA-4 and PD-L1 in cancers are rather limited. Indeed, recent findings by Rouas-Freiss et al. (75) revealed that intra- and inter-tumor heterogeneity of PD-1/PDL-1 and HLA-G/ILT2/ILT4 expression does exist in various areas of the same lesion or among different renal-cell carcinoma lesions and in tumor infiltrating immune cells. In this regard, blocking multiple checkpoints is urgently needed to target the entire tumor.

In addition to the complexity of the tumor itself, heterogeneous tumor immune microenvironments including varying profiles of immune checkpoints molecule expression can lead to different responses to immune therapy. In this context, MHC I and II molecule expression pattern in melanoma patients were reported to confer differential sensitivity to CTLA-4 and PD-1 blockade therapy. Data revealed that an anti-CTLA-4 therapy response requires MHC class I, while an anti-PD-1 response is associated with MHC class II expression (76). However, the significance of HLA-G expression for anti-CTLA-4 and/or anti-PD-1 therapy needs to be evaluated. Given this evidence, the proposed multiple checkpoint blockade to overcome resistance to therapy and relapse should take MHC expression status into consideration (75, 77).

## HLA-G heterogeneity in cancers

Genetic and proteomic heterogeneity regarding markers such as HLA-G is a common phenomenon in tumors. The inter- and intra-tumor heterogeneity of HLA-G expression can be increased by the complexity of mechanisms involved in the regulation of HLA-G expression (20, 75). In addition to the genetic background of HLA-G, multiple transcriptional, epigenetic, post-transcriptional and environmental mechanisms and contexts are involved in modulating HLA-G mRNA and/or protein expression (13, 78). In this context, a number of HLA-G specific microRNAs, including the miR-152 family (miR-148a, miR-148b, miR-152) and miR-133 have been demonstrated to control HLA-G expression (39, 79). Furthermore, cytokines such as IFN-γ and/or TNF-α differentially regulated HLA-G isoform expression in a retinal pigment epithelial cell line (80). In pathological settings, fewer functional HLA-G molecules and differential expression of HLA-G isoforms were observed in asthma and prostatic adenocarcinoma patients, respectively (81, 82). All these mechanisms of regulation and disease status alone or in combination can contribute to the heterogeneity of HLA-G expression.

## Intratumor HLA-G heterogeneity

Intratumor heterogeneity is very common in cancers; however, information on intratumor HLA-G heterogeneity is rather limited. In a cohort of nineteen clear cell renal-cell carcinoma patients (ccRCC), normal adjacent tissues and tumor lesions were collected for each patient. Among these samples, different tumor areas including 3–4 zones per tumor were obtained, and HLA-G expression was evaluated with immunohistochemistry. The authors found that intratumor HLA-G heterogeneity was present in all the ccRCC patients; levels of HLA-G expression varied markedly among different areas. In some patients, a high degree of HLA-G expression was observed in all selected areas; however, in other patients, HLA-G expression was only found in some but not all areas among CA9^+^ ccRCC tumor cells (75).

In another study, expression patterns of HLA-G isoforms in ccRCC lesions were analyzed with next-generation sequencing technologies and immunohistochemical labeling (20). In this in-depth study, expression levels of HLA-G isoforms such as HLA-G1, -G5, and -G6 were highly variable among tumors from different patients (intertumor heterogeneity) and among distinct areas or subcellular locations in the same tumor (intratumor heterogeneity). Importantly, the authors also identified novel HLA-G isoforms that had not been recognized before, such as the predicted isoforms without a transmembrane region and α1 domain. However, mechanisms underlying the high diversity of HLA-G expression and whether they involve a manner of spatial separation or selection of subclones remain to be explored.

## Intertumor HLA-G heterogeneity

### Interpatient intertumor HLA-G heterogeneity

Aberrant HLA-G expression in cancers was first reported in 1998 (11), and studies later documented that up-regulated HLA-G expression could only be found in primary and metastatic melanoma cells; but not in tumor regression sites or in normal adjacent skin tissues from a melanoma patient (83, 84). Based on these pioneering studies, the hypothesis that HLA-G expression is associated with malignant transformation and tumor cell immune evasion was proposed.

Since Paul's first report (11), HLA-G expression has been analyzed and evaluated worldwide in thousands of malignant samples of various types of cancers (12). Aberrant HLA-G expression in cancers has been found to be associated with advanced tumor stage, metastasis status and poor disease outcome. However, among and within different tumor types, discrepancies regarding HLA-G expression profiles have been observed in various types of cancers, such as breast cancer (85–94), colorectal cancer (CRC) (95–100), cervical cancer (101–103), endometrial cancer (104, 105), oesophageal squamous cell carcinoma (ESCC) (106–108), Ewing sarcoma (109), gastric cancer (110–112), glioblastoma (113), HCC (49, 114, 115), lung cancer (116–118), lymphoma (119–122), nasopharyngeal carcinoma (123), oral squamous cell carcinoma (124), ovarian cancer (125–127), pancreatic adenocarcinoma (128–130), and thyroid cancer (131, 132). The details of the methods applied and main conclusions drawn from these studies are summarized in Supplementary Table 2.

Notably, the percentage of HLA-G expressing malignant cells depends on the type of cancers, and expression levels vary from negative to totally positive among different types of cancers (133, 134). However, the degree of HLA-G expression also varies dramatically among different laboratories for almost every histological type of tumor studied. For example, HLA-G expression was assessed by immunohistochemistry with the same HLA-G monoclonal antibody (mAb), 4H84, in seven previous studies on breast cancer, in which the percentages of HLA-G expression ranged from 24 to 94.1% (91, 94). In four other studies using the HLA-G mAbs MEM-G1 (92), MEM-G2 (90), HGY (87) and 5A6G7 (89), the percentages of HLA-G positive staining cells were 43.8, 62.2, 66, and 59.6%, respectively. Similarly, rather low interlaboratory concordance for HLA-G expression has been observed for other malignancies.

The low concordance between different laboratories for the same type of tumor lesions may reflect differences between assessment methods and tumor heterogeneity. Indeed, among these studies, there is a wide variation of immunohistochemistry protocols, including variations in the antibodies used and their dilution, the incubation time and staining evaluation criteria. Different cohort sizes and clinicopathological parameters such as treatment history, tumor subhistological type and tumor immune microenvironment could all affect the evaluation of the status of HLA-G expression among cancer patients.

Furthermore, genetic backgrounds of HLA-G may also influence the profile of HLA-G expression; for example, in the case of HLA-G molecules generated by the primary transcripts from two *HLA-G* null alleles. One null allele is the HLA-G^*^0105N (135) with a cytosine deletion at position 1597 (_Δ_C) in exon 3, which causes an open reading frame shift mutation and generates a premature stop at either codon 189 (TGA) in exon 4 or at codon 297 (TAG) in exon 5. As a result of this mutation, the translation and expression of the α2 domain-containing HLA-G isoforms, including HLA-G1, -G4, and HLA-G5, are disrupted, while the functional isoforms HLA-G2, -G3, -G6, and HLA-G7 can be still normally expressed (136, 137). The other null allele, HLA-G^*^01:21N (138), is a nucleotide mutation at codon 226 (CAG) that results in an early stop codon (TAG) in exon 4 and, is predicted to translate a truncated and presumably non-functional protein.

### Intrapatient intertumor HLA-G heterogeneity

This heterogeneity is noticeable in a patient with multiple tumors of the same type. In cervical cancers, Ferns et al. (139) analyzed HLA-A, -B, -C, HLA-E and HLA-G expression on primary tumors and case-matched lymph node (LN) metastases by immunohistochemistry. In a cohort of 136 patients, HLA-G expression (probing with mAb 4H84) was detected in 25% of the primary cervical cancers and in 11% of paired metastatic LNs. In squamous cell carcinoma (SCC) subtypes, HLA-G positive staining was found in 22% (20/90) of the primary tumors and 20% (18/90) of their LN metastases. In adenocarcinoma (AC), positive HLA-G was staining observed in 31% (10/32) of the primary tumors and in 28% (9/32) of paired LN metastases. These findings revealed that primary tumors and paired LN metastases in both histology types of cervical cancers had similar HLA-G expression patterns. Similarly, a study by Guimarães et al. (103) revealed that, in invasive cervical cancer (ICC) patients, HLA-G5 expression was positive in 31.6% (25/79) of the patients. Among these patients, HLA-G5 expression was observed in 29.6% (8/27) of the patients with LN metastasis and in 32.7% (17/52) patients without LN metastasis.

An evaluation of HLA-G expression in primary CRC and liver metastases using different HLA-G mAbs including 4H84, MEM-G/1 and MEM-G/2, was performed by Swets et al. (95). In this study, HLA-G expression was observed in 29, 6, and 10% of primary CRC lesions, and 30, 4, and 0% of paired liver metastases when using the mAbs 4H84, MEM-G/1 and MEM-G/2, respectively. These findings indicated that HLA-G molecule probing with different antibodies with different specificities could dramatically impact the interpretation of the relevance of HLA-G in clinical settings.

### HLA-G heterogeneity and cancer therapy

Alterations in HLA expression are frequent and early events during tumor development and progression as a result of immune editing (140). Consequently, tumor cells that fail to present surface neoantigens due to an altered classical HLA class I complex can avoid killing by T cells, and aberrant induction of HLA-G expression can suppress the functions of various immune cells for immune escape (141).

As with other molecules, mechanisms underlying the regulation of HLA-G expression involve in multiple levels such as epigenetic modification, transcriptional regulation and post-transcriptional modifications (78, 79). In addition, microenvironmental factors such cytokine profiles and even therapeutics can enhance HLA-G expression. In this context, previous studies showed that HLA-G expression could be induced by 5-aza-2′-deoxycytidine and IFN-γ treatment *in vitro* in glioblastoma, and an enhanced peripheral sHLA-G level was observed in melanoma patients treated with IFN-α (113, 142). Given the immune suppressive functions of HLA-G in favoring tumor immune evasion and progression, induction of HLA-G expression during immune therapy which may impair the therapeutic effects, should be considered.

Moreover, the fact that HLA-G expression status can dramatically affect therapeutic responses has been addressed in tumor patients and patients with other diseases. In 2000, Wagner et al. (15) highlighted the importance of classic and non-classic HLA molecule expression status in melanoma patients treated with postsurgery adjuvant IFN-α-2b. In that study, they found that the status of classic HLA I molecules and HLA-G expression were significantly related to the outcome of melanoma patients receiving IFN-α-2b treatment. Disease relapse occurred in patients with HLA-G expression that had lost classic HLA I molecules. However, with or without HLA-G co-expression, no relapse was observed in patients with classic HLA I molecule expression. This study indicated that HLA-G expression can enable classic HLA I deficient melanoma cells to lose responsiveness to IFN-α treatment. Thus, evaluation of the status HLA antigen expression before immunotherapy may have important practical implications for treatment.

## Conclusion

Tumor heterogeneity has gained attention in the field of individualized cancer therapy due to its implications for oncogenesis, and metastasis, and its potential to affect responses to therapies and clinical outcomes (3). Tumor heterogeneity can be caused by genetic mutations during clonal evolution, and/or by tumor microenvironment selective pressure during tumor development (143).

Alteration of HLA antigen expression is a well-recognized mechanism employed by malignant cells to avoid innate and adaptive immune surveillance and responses (144). Many efforts have been made by multiple laboratories worldwide to assess the expression and clinical significance of HLA-G expression in cancers. From these studies, a strong relationship of HLA-G expression with tumor progression and patient outcomes has been established.

Based on numerous previous studies, HLA-G has been promoted as a new checkpoint molecule and a promising immunotherapy target, such as via application of HLA-G antagonists or anti-HLA-G or anti-ILT antibodies to block the interaction between ILT receptors and HLA-G. Additionally, HLA-G as an effective target for drug delivery was proposed by Zhang et al. (145). In that study, authors successfully developed HLA-G antibody and methotrexate (MTX)-loaded nanobubbles (mAb_HLA−G_/MTX/PLGA NBs) as HLA-G-targeted drug delivery molecule. The results showed that mAb_HLA−G_/MTX/PLGA NBs could specifically transport to the HLA-G positive tumor cells *in vitro* or tumor tissues *in vivo* in a murine model, and the released MTX from the NBs could kill the residual tumor cells and inhibit the reoccurrence of tumors.

However, available results from the literature reveal that intertumor and intratumor heterogeneity of HLA-G expression varies dramatically. In addition to the heterogeneity that inherently occurs in tumors, the low concordance in HLA-G detection and evaluation results among different laboratories remains a major obstacle for the interpretation of the clinical significance of HLA-G. These controversies might be caused by the use of different HLA-G monoclonal antibodies, technical procedures, immunostaining evaluation criteria, or different clinicopathological and HLA-G genetic backgrounds of the included cohorts. Therefore, international recommended standardization protocols, larger cohorts and prospective studies are required to confirm and validate HLA-G as a target before routine clinical application. Furthermore, in addition to the seven well-known HLA-G isoforms, novel unrecognized HLA-G isoforms do exist, as presented by Dr. Carosella and colleagues (20). In this context, more specific antibodies for a particular HLA-G isoform and new technologies such as next generation sequencing are needed. With these tools, we can further discriminate different isoforms and evaluate their respective functions in tumor biology to finally improve and optimize personalized medicine.

## Author contributions

All authors listed have made a substantial, direct and intellectual contribution to the work, and approved it for publication.

### Conflict of interest statement

The authors declare that the research was conducted in the absence of any commercial or financial relationships that could be construed as a potential conflict of interest.
