# Supplementary material for: Heterogeneity of HLA-G Expression in Cancers: Facing the Challenges
Source: Front Immunol. 2018 Sep 27;9:2164. doi: 10.3389/fimmu.2018.02164 (PMC6170620; doi:10.3389/fimmu.2018.02164)
Supplement: Supplementary file 1 [file Table_1.PDF]

Suppl. Table 1. Currently available immunohistochemistry antibodies for HLA-G detection

HLA-G mAbs	Specificity	Applications	Immunogen
4H84 (IgG1)	An epitope in the α1 domain	IHC(P), IP, WB, ICC, ELISA,	Amino acids 61-83 of HLA-G α1 domain of human origin
01G (IgG1)	*mHLA-G (full-length HLA-G1), but not sHLA-G isoforms	IHC(F), IP, ICC, FC, ELISA,	HLA-B27 transgenic mice imunized with H-2 identical murine cells transfected with HLA-G and human β2-microglobulin
87G (IgG2a)	Both mHLA-G and sHLA-G (HLA-G1 and HLA-G5). 87G blocks interaction of HLA-G with inhibitory receptors	IHC(F), FUNC, FC, ELISA	
MEM-G/1 (IgG1)	Denaturated HLA-G heavy chain	IHC(F/P), WB	Denatured bacterially expressed recombinant human HLA-G heavy chain
MEM-G/2 (IgG1)	Free heavy chain of all HLA-G isoforms		
MEM-G/9(IgG1)	Native form of HLA-G1 and HLA-G5 isoform associated with β2-microglobulin. Reactivity with HLA-G3 was reported.	IHC(F), IP, ELISA, FC,	Recombinant human HLA-G refolded with β2-microglobulin and peptide
MEM-G/11(IgG1)	HLA-G1 antigen	IHC(F), IP, ELISA, FC, ICC	
2A12 (IgG1)	HLA-G5 and -G6 isoforms	IHC(F/P), WB, FC, ELISA,	C-terminal amino acid sequence (22-mer) of HLA-G5 and HLA-G6 proteins coupled to ovalbumin
5A6G7 (IgG1)	HLA-G5 and -G6 isoforms	IHC(F/P), WB, FC, ELISA, ICC	

*mHLA-G:membrane-bound HLA-G isoforms; sHLA-G:soluble HLA-G isoforms
