# Supplementary material for: Heterogeneity of HLA-G Expression in Cancers: Facing the Challenges
Source: Front Immunol. 2018 Sep 27;9:2164. doi: 10.3389/fimmu.2018.02164 (PMC6170620; doi:10.3389/fimmu.2018.02164)
Supplement: Supplementary file 2 [file Table_2.PDF]

Suppl. Table 2. Intertumor HLA-G expression in cancers

Cancer types	Sample size	Methods (Ab)	HLA-G (%)	Immuno-staining evaluation	Main conclusions of HLA-G expression	Ref.
Breast cancer	39	IHC (4H84)	41%	Negative (0), 1% ~ 5% (1), 6% ~ 25% (2), 26% ~ 75% (3), and 76% ~ 100% (4).	Associated with shorter disease-free survival.	85
	58	IHC (4H84)	70.7%	Negative (0), 1% ~25% (1), 26% ~ 50% (2), 51% ~ 75% (3), and >75% (4).	Associated with advanced disease stage.	86
	235	IHC (HGY)	66%	Negative (-), < 25% (+) and/or weakly, 25% ~ 50% and/or moderately (++), and > 50% and/or strongly stained (+++) .	An independent prognosis factor.	87
	501	IHC (4H84)	60%	Any staining of tumor cells as positive and no staining as negative.	A prognostic factor among classical HLA class I negative patients,.	88
	52	IHC (5A6G7)	59.6%	Negative (< 25% positivity) and positive (>25% positivity) .	Associated with aggressiveness.	89
	45	IHC (MEM-G/2)	62.2%	Positive when ⩾15% of staining.	Associated with shorter survival.	90
	102	IHC (4H84)	94.1%	Negative (-), weak staining (+); moderate staining (++) and strong staining (+++).	HLA-Glow is associated with higher overall and relapse-free survival rates.	91
	73	HC (MEM-G/1)	43.8%	Positivity when ⩾25% of staining, irrespective of staining intensity .	Not associated with clinical parameters	92

Suppl. Table 2. Intertumor HLA-G expression in cancers—cont'd

Cancer types	Sample size	Methods (Ab)	HLA-G (%)	Immuno-staining evaluation	Main conclusions	Ref.
Breast cancer	88	IHC (4H84)	25%	No details described.	Not detected in low-grade ovarian serous carcinomas.	93
	2042	IHC (4H84)	24%	Any staining of tumor cells considered positive.	Not associated with clinical outcome.	94
CRC	81	IHC (4H84) (MEMG/1) (MEM-G/2)	29% 35% 19%	Based on presence or absence of positive stained cells.	HLA-G expressed in majority of the primary tumors but not in associated liver metastasis.	95
	201	IHC (HGY)	64.6%	Without staining (-), < 25% and/or weakly (+), 25% ~ 50% and/or moderately (++) and > 50% and/or strongly stained (+++).	An independent prognosis factor.	96
	102	IHC (MEM-G/2)	70.6%	Based on presence or absence of positive stained cells.	Associated with worse survival.	97
	457	IHC (4H84)	70.7%	HLA-G positive when >5%, irrespective of staining intensity.	HLA-G expression > 55% associated with worse prognosis.	98
	285	IHC (4H84)	22.1%	Intensity of staining (absent, weak, moderate or strong).	Associated with worse survival and DFS.	99
	484	IHC (4H84)	27.7%	Intensity of staining (absent or faint in <20%), weak (faint to weak in >20% but ≤70%), moderate (weak to moderate in >70%) or strong (intense in 20% ~70%).	associated with presence of Foxp3+ cells.	100

Suppl. Table 2. Intertumor HLA-G expression in cancers—cont'd

Cancer types	Sample size	Methods (Ab)	HLA-G (%)	Immuno-staining evaluation	Main conclusions	Ref.
Cervical cancer	58	IHC (5A6G7)	75.86%	No expression (0), 1% ~30% (1), 31% ~70% (2), 71% ~100% positive cells (3).	An early marker for lesion progression.	101
	143	IHC (4H84)	60%	Membrane or combined membrane and cytoplasmic expression of HLA-G were interpreted as positive.	Associated with disease progression.	102
	79	IHC (5A6G7)	31.6%	Low expression when no signal or discrete staining; high expression when moderate or intense staining.	HLA-G detected in 17 (32.7%) without and 8 (29.6%) with metastasis.	103
Endometrial carcinoma	44	IHC (4H84)	55%	Negative (0), 1%~5% (1), 6%~10% (2), 11%~25% (3), 26%~50% (4) and >50% (5).	Associated with disease stage.	104
	525	IHC (4H84)	39.8%	Negative (0); 1%~5% (1); 5%~25% (2); 25%~50% (3); 50%~75% (4) and >75% (5). The intensity scored (0: absent, 1: weak, 2: moderate, 3: strong) . The sum of both scores. A score of ≥2.5 considered as up-regulation of HLA-G.	Not associated with survival.	105
Esophageal cancer	121	IHC (HGY)	90.9%	Without staining(−);< 25% and weakly (+);25%~50% and moderately (++);> 50% and strongly stained (+++).	An independent prognosis factor.	106
	79	IHC (4H84)	65.8%	HLA-G expression was graded as: negative, 1%~25% (1+), 26%~50% (2+), 51%~75% (3+) and >75% (4+)., irrespective of staining intensity.	HLA-G is an independent prognosis factor.	107
	60	IHC (MEM-G/1)	70%	Without staining(0); <25% (1+); 25%~50% (2+); and >50% (3+). Nnegative and 1+ as HLA-G negative, 2+ and 3+ as HLA-G positive.	Associated with cancer cell differentiation, lymph node metastasis.	108

Suppl. Table 2. Intertumor HLA-G expression in cancers—cont'd

Cancer types	Sample size	Methods (Ab)	HLA-G (%)	Immuno-staining evaluation	Main conclusions	Ref.
Ewing sarcomas	47 (primary)<br>12 (relapse)	IHC (4H84)	30%<br>33%	Graded by low, intermediate or strong densities.	Associated with tumor infiltrating T cells.	109
Gastric cancer	160	IHC (HGY)	71%	Without staining(-); <25% and/or weakly (+);with 25%~50% and/or moderately (++); >50% of the cancer tissues and/or strongly stained (+++).	An independent prognosis factor.	110
	52	IHC (5A6G7)	31.%	HLA-G positivity when >10%.	An independent prognosis factor.	111
	179	IHC (4H84)	49.7%	Negative; 1%~25% (+); 25%6~50%(++); >50% (+++).	An independent prognosis factor.	112
Glioblastoma	108	IHC (MEM-G/2)	60.2%	No details described.	HLA-G-negative patients were alive longer than HLA-G positive patients.	113
Hepatocellular carcinoma	173	IHC (MEM-G/1)	low (43%) high (57%)	The density of HLA-G staining evaluated with computerized image system.	Associated with poor survival and increased recurrence;	114
	36	WB (MEM-G/1)	66.7%	No details described.	An independent prognosis factor.	115
	219	IHC (4H84)	50.2%	Negative, and positive grouped as 1%~25%, 26%~50%, 51%~75% and >75%.	Associated with advanced disease stage.	49
Lung cancer	106	IHC (HGY)	75%	Without staining (−);< 25% and weakly (+);25%~50% and moderately (++);>50% and strongly stained (+++).	An independent prognosis factor.	116

Suppl. Table 2. Intertumor HLA-G expression in cancers—cont'd

Cancer types	Sample size	Methods (Ab)	HLA-G (%)	Immuno-staining evaluation	Main conclusions	Ref.
Lung cancer	101	IHC (4H84)	41.6%	Negative (0), 1%~25% (1), 26%~50% (2) and >50% (3), irrespective of staining intensity.	Associated with advanced disease stage.	117
	131	IHC (5A6G7)	34%	Negative≤5% and positive >5%.	Predominately expressed in adenocarcinoma.	118
Lymphoma (classical Hodgkin)	175	IHC (MEM-G/1)	54%	Positive when > 50% of neoplastic cells showed stronger staining.	Associated with absence of MHC class I expression on HRS cells and EBV negative status.	119
	20	IHC (4H84)	55%	Negative staining (0), <25% (1), 26% ~ 50% (2), 51% ~ 75% (3), 76% ~ 100% (4).	Different patterns of HLA-G expression associated with different outcomes.	120
Lymphomas (Diffuse Large B-Cell)	148	IHC (4H84)	24%	Positive when >25% of lymphoma cells expressed intermediate/strong staining.	Negative HLA-G expression associated with worse survival.	121
Lymphomas (cutaneous T- and B-cell)	45	IHC (4H84)	51%	HLA-G positivity as a strong (numerous cells) or as a single-cell positivity (scant, scattered cells throughout the infiltrate).	Associated with high-grade histology and advanced stage in CTCL.	122
Nasopharyngeal carcinoma	552	IHC (4H84)	79.2%	Intensity as (neg), weak (1), moderate (2) or strong (3). Percentage <5% (0); 5%~25% (1); 26%~50% (2); 51%~75% (3); 76%~100%(4). A score by adding intensity and positive cells.	Associated with poor prognosis, recurrence or metastasis.	123
Oral squamous cell carcinoma	60	IHC (MEM-G/2)	50%	An immunoreactive score (IRS) calculated by multiplying the percentage and staining intensity. IRS=0 (negative), <2 (low), >2 (high).	Lower HLA-G expression associated with longer survival.	124

Suppl. Table 2. Intertumor HLA-G expression in cancers—cont'd

Cancer types	Sample size	Methods (Ab)	HLA-G (%)	Immuno-staining evaluation	Main conclusions	Ref.
Ovarian cancer	40	IHC (4H84)	low (55%) moderate (20%) strong (25%)	0% ~ 25% stained tumors and mild staining (1+); 25% ~ 50% and moderately staining (2+); >50% and strongly staining(3+).	HLA-G expression >17% associated with poor survival.	125
	34	IHC (MEM-G/2)	35%	No details described.	Associated with high-grade histology.	126
	118	IHC (5A6G7)	79.7%	Percentage of stained cells >5% (+),<5% (-).	Not associated with clinical parameters.	127
Pancreatic adenocarcinoma	122	IHC (Rabbit polyclonal Ab)	low (36.1%) high (63.9%)	Proportion (0, none; 1,⩽25%; 2, 26%~50%; 3, >50%). Intensity (0, none; 1, weak; 2, moderate; 3, strong).	An independent prognosis factor.	128
	42	IHC (4H84)	66%	1%~25% (negative), 26%~50%, 51%~75% and >75%, irrespective of staining intensity.	Associated with advanced stages	129
	158	IHC (not described)	39.2%	Negative: <5%; local: 5%~75%; diffuse: >75%, irrespective of staining intensity.	Associated with worse survival.	130
Thyroid carcinoma	138	IHC (5A6G7)	90.6%	Without staining (–); < 25% (+); 25%~50% (++); and > 50% of cell staining (+++).	Associated with poor prognosis	131
	70	IHC (MEM-G/2)	44.3%	No details described.	Associated with lymph node metastasis.	132
